# Clinical Characteristics of Torsion of the Omentum

**DOI:** 10.4021/gr2009.07.1303

**Published:** 2009-07-20

**Authors:** Alvaro Montiel-Jarquin, Aurelio Lopez-Colombo, Arnulfo Nava, Rayo Juarez-Santiesteban, Hugo Leyva-Trejo, Alfonso Zamora-Ustaran, Mario García-Carrasco, Margarita Munoz-Guarneros

**Affiliations:** a Gastroenterologist and General Surgeon, at Instituto Mexicano del Seguro Social in Puebla, Mexico; b Research Department, Instituto Mexicano del Seguro Social, Puebla and Benemérita Universidad Autónoma de Puebla, Mexico

**Keywords:** Torsion of the greater Omentum, Clinical characteristics, Retrospective study

## Abstract

**Background:**

The aim of this paper is to describe clinical aspects of the torsion of the omentum.

**Methods:**

In this observational, retrospective study, the study group consisted of patients surgically managed for torsion of the omentum, between 1998 and 2008, in a second level medical facility in Mexico. Variables in the study included age, sex, signs and symptoms, body mass index (BMI), treatment and evolution time. Descriptive statistical analysis was employed.

**Results:**

Eleven patients were confirmed torsion of omentum, 7 (63.63%) women and 4 (36.36%) men, median age 33 (20 to 58) years, BMI > 25.0 in 9 (81.81%), average evolution 6.54, SD 3.47 days. All presented with abdominal pain, 6 (54.54%) with abdominal distension, 4 (36.36%) with ambulatory difficulty, 3 (27.27%) with malaise, and 5 (45.45%) with previous surgery. In all cases diagnosis was made by means of laparotomy, treatment was the resection of the affected segment, and there were no further complications.

**Conclusions:**

Torsion of the omentum resembles acute appendicitis; abdominal pain and abdominal distension are the most common symptoms. It is often discovered during surgery and it is treated surgically by removal of the affected segment of the omentum.

## Introduction

Torsion of the omentum is a rare pathology, and its clinical presentation mimics acute appendicitis [1]. The first case of torsion of the omentum was described by Bush in 1896. By 1908 approximately 112 cases had been described [2], and in 1991 Coppo gathered data on nearly 150 cases [3, 4]. The first cases of omentum pathology at the Hospital General Regional 36, at the Instituto Mexicano del Seguro Social in Puebla, Mexico, were reported in 1998 [5] and 2004 [6]. By 2001, slightly fewer than 300 cases had been reported, 85% of them in the adult population and the remaining 15% in the pediatric population, almost all diagnosed as acute appendicitis and discovered during an exploratory laparotomy [1].

In children, 0.05 to 0.1% of cases are diagnosed during a laparotomy for acute appendicitis[1, 7]. The condition is more common in males, with a ratio of 2-5.1 [1, 8], in the third and fourth decades of life, but it can occur at any age[5, 6]. It can be primary or idiopathic, when no underlying cause or associated factors are found; or secondary, when the cause is identified [1, 9-11]. Primary torsion is less common than secondary [1]. Longer or more swollen than normal omentum, internal hernias, inflammatory pathologies of other organs such as acute cholecystitis, pancreatitis, and adnexitis, tumors, and postsurgical adhesions are causes of secondary torsion of the omentum [1]. Its etiology remains associated with the predisposing factors such as sex, obesity, sudden strong increase in intra-abdominal pressure brought on by coughing or violent exercise, traumas, autonomics (large pedicle), larger or more twisted than normal epiploic blood vessels, accelerated peristalsis, surgical adherences, or some acute process in an intracavitary organ that causes the migration of a segment of the omentum to the affected site, resulting in torsion[5, 6, 9]. Torsion of the omentum presents with light abdominal pain that is similar to acute appendicitis or less similar to any other abdominal surgical pathology. However, the evolution of symptoms and clinical signs is slower and less intense, which creates a delay in the time it takes for the patient to seek medical assistance[10, 11]. The diagnosis is frequently made during exploratory laparotomy [1, 5, 6]. However, ultrasound and computed axial tomography are useful tools for making a preoperative diagnosis [12-16]. Torsion of the omentum is considered a cause of right lower quadrant pain (RLQP) and acute abdomen [5].

When omental torsion is present, edema and the inflammatory process make the clinical presentation to progress to necrosis of the twisted segment, more frequently on the right side due to the length and characteristics of the greater omentum [8, 11]. The treatment consists in the removal of the affected segment either by means of laparoscopy or laparotomy, with excellent results [17-20].

The aim of this paper is to present the clinical characteristics of patients with torsion of the omentum, treatment, and evolution.

## Methods

An observational and retrospective review was conducted of all cases of torsion of the omentum in a second level medical facility in Puebla, Mexico. The study included all patients who were operated on for torsion of the omentum, between January 1, 1998 and December 31, 2008.

Volume 4-30-27/90 (Surgical Record) was consulted to locate clinical records for the study. The study variables were age, sex, body mass index (BMI), time of evolution from the onset of symptoms to surgical intervention, preoperative and postoperative diagnosis and evolution. The BMI was interpreted according to Quetelet´s Index: 18 to 25 healthy; above 25 overweight; above 30 somewhat obese; and above 40 morbidly obese. Descriptive statistical analysis was employed.

## Results

A total of 112,830 surgical procedures (January 1, 1998 to December 31, 2007) were performed (source: Unique Information System database (UISD) in the Puebla State Regional Office of the Instituto Mexicano del Seguro Social, of these, 11 patients had torsion of the omentum. Seven (63.63%) were women and 4 (36.36%) were men, with a median age of 33 years (20 to 58); hyperthermia was present in 4 (36.36%) patients; the average BMI was 29.06 kg/m^2^ (SD 2.76); 2 patients (18.18%) had a BMI < 24.91 kg/m^2^ and 9 (81.81%) had a BMI > 25kg/m^2^; the average duration of clinical presentation of symptoms was 6.54 (SD 3.47) days. The signs and symptoms of the patients are shown in Table 1. The predisposing factors, cause of lesion, and surgery performed of the cases are shown in Table 2. No patients’ abdomens were examined by means of ultrasound or computed axial tomography.

**Table 1:** Signs and symptoms of patients with torsion of the omentum

Signs and symptoms of patients with torsion of the omentum	n= 11	%
Constipation	3	27.27
Malaise	3	27.27
Fever (> 38.2 °C)	4	36.36
Vomiting	4	36.36
March impairment	4	36.36
Abdominal distention	6	54.54
Pain	11	100

**Table 2:** Clinical Characteristics of patients with torsion of the omentum

n	Sex	Age (Years)	BMI	Previous Surgery	Duration (Days)	Location of pain	Cause of lesion
1	F	20	28.84	Caesarian	6	RLQ	X
2	F	33	32.02	None	4	RLQ	X
3	F	58	29.74	Appendect.	2	RUQ	CCC
4	M	26	30.22	None	12	RLQ	X
5	F	32	30.26	None	8	RLQ	TROC
6	M	41	29.49	Appendect.	5	RLQ	X
7	F	33	31.98	None	9	RLQ	X
8	F	23	23.33	None	12	RLQ	X
9	F	41	30.85	Appendect	2	RLQ	X
10	M	36	32.81	None	5	RLQ	X
11	M	28	30.47	Appendect	7	RLQ	X

BMI= body mass index, RLQ= right lower quadrant, RUQ= right upper quadrant, X= none, CCC= chronic calculous cholecystitis, TROC= twisted right ovarian cyst.

In 7 (63.63%) patients the preoperative diagnosis was acute appendicitis and the postoperative was confirmed in all cases by histopathologic study as torsion of the omentum. In a year follow up, none presented any complication.

## Discussion

Torsion of the omentum is a rare cause of acute abdomen. Its presentation mimics acute appendicitis and other pathologies that cause acute abdomen. In this study group, females were more commonly affected, although most authors report predominance in men [1, 4, 8], and in some groups no predominance by sex is mentioned [1]. The reported incidence ranges between 0.16 and 0.37. The duration of the clinical presentation of symptoms in this group is greater than that of acute appendicitis, probably due to the fact that the intensity of symptoms is less. This concurs with reports from other groups [1].

Pain is the predominant symptom, and its location depends on the affected site of the omentum, in literature it is reported in 100% of the cases, rarely occurs in the upper right quadrant of the abdomen and is continuous. The hyperthermia that was present in 36.36% of patients was less than 38.2° C, which does not concur with information from some authors who report temperature elevations up to 39.5°C [1].

Previous surgery was a predisposing factor in 5 (45.45%) of the patients with a BMI greater than 25. 7 (63.63%) patients had a BMI greater than 30, which coincides with data in world literature that identifies obesity as a predisposing factor for torsion of the omentum [1, 5, 6, 9].

In 2 (18.18%) patients the cause of the torsion was identified: one (9.09%) with an intensifying chronic calculous cholecystitis that caused pain in the upper right quadrant of the abdomen and one (9.09%) with a twisted right ovarian cyst. The acute inflammatory process in these organs caused the omentum to migrate, resulting in its torsion.

As in other study groups, all the patients underwent surgery for acute abdomen. The definitive diagnosis was obtained by means of exploratory laparotomy. In the cases where there was no preceding appendectomy, the preoperative diagnosis was probable acute appendicitis due to the similarity of symptoms. As in the majority of published studies, no preoperative diagnosis was made in any of the cases in this group [21].

The imaging data from simple x-rays of the abdomen showed the presence of a fixed, air-filled small bowel loop in 6 (54.54%) patients, an indication that suggested surgical acute abdomen. Although some authors report that therapeutic diagnostic laparoscopy can be very useful for diagnosing and managing segmental torsion of the greater omentum [15, 17-20], the majority of cases reported in the literature have been diagnosed by laparotomy.

Treatment consists of removing the affected segment of the greater omentum, as well as managing concomitant pathology when it exists [16, 18, 19]. Conservative management in patients without associated complications has also been reported [22]. In this study group, treatment by means of laparotomy achieved good results without complications. However, treatment by laparoscopy can achieve a better aesthetic result and lessen the time of hospitalization [19, 20].

In conclusion, torsion of the omentum is a rare pathology. Its clinical presentation is similar to acute appendicitis or to any other cause of surgical acute abdomen. It has a tendency to present in obese people. Pain and abdominal distention are the predominant symptoms. For the most part, the condition is idiopathic, which makes it difficult to identify the cause in up to 33.3% of cases. Preoperative clinical diagnosis is difficult, and it must depend on complementary studies when they are available. Management of the condition is surgical, partial omentectomy and treatment of the original cause of the torsion. The evolution is good when correct treatment is applied, even when delayed.
